# NEWS

**DOI:** 10.7189/jogh.03.020203

**Published:** 2013-12

## DEMOGRAPHY

• In 2008, for the first time, half the world’s population lived in cities. Studies show that 25% of the world’s population live in 600 cities that generate up to 60% of global output. In 1975, there were just three megacities (New York, Mexico City and Tokyo), with populations of over 10 million. Today, there are at least 20. Many of the world’s biggest cities are vulnerable to natural disasters eg, flooding and earthquakes. Their rapid growth in developing countries means that many of their citizens live in vast slums. By 2025, it is estimated that only four megacities will be in Europe or North America – the remainder will be in Africa, Asia and Latin America. City governments are often weak, with limited clout to deal with their problems. “These megacities are a big part of humanity’s future. The prospect should be both exhilarating and terrifying – and a call to action for better urban policies,” says Harvard University’s Edward Glaesar, author of *Triumph of the City.* (*Financial Times*, 1 Feb 2013)

• The 50th anniversary of the African Union’s (AU) establishment in 1963 is an opportunity to review its strengths, weaknesses, and achievements. Its mission is to promote pan–African political self–determination, economic self–reliance and solidarity. It was steadfast against white minority rule and colonialism, launching Africa’s path to decolonisation and majority rule. This culminated in the peaceful dismantling of apartheid in South Africa in 1994. It has mediated in border disputes to resolve conflicts – a major cause of interstate conflict. However, the AU has been less likely to intervene in state–sponsored terrorism and heinous crimes, including ethnic cleansing and genocide. Today, the AU is lauded for deploying peace–keeping forces, and playing a greater role in resolving internal conflicts. Its Regional Economic Communities lead peace–keeping initiatives, and promote economic co–operation and integration. It is hampered by a lack of resources, and is heavily dependent on external funding. This raises questions about African ownership. Its internal structures risk spreading its influence too thinly, and it is criticised for its membership, which includes countries with poor human rights records and electoral fraud. (*The Guardian*, 21 May 2013)

• A UN report predicts that the world population will rise from the current figure of 7.2 billion to 9.6 billion in 2050. It states that the population in developed countries should remain static, whilst the birth rates in developing countries are predicted to soar. The Director of the Population Division in the UN’s Department of Economic and Social Affairs, Mr John Wilmoth, said that “rapid growth is expected to continue over the next few decades in countries with high levels of fertility such as Nigeria, Niger, the Democratic Republic of the Congo, Ethiopia and Uganda but also Afghanistan and Timor–Leste, where there are more than five children per woman.” The report analysed demographic data from 233 countries. It also predicted an increase in life expectancy, with people living up to 89 years in developed countries, and up to 81 years in developing countries by 2100. (*UN News*, 13 Jun 2013)

• The UN High Commissioner for Refugees gave a stark warning that the world has the worst refugee crisis since the Rwandan genocide of 1994. Its global trends report stated that more than 45.2 million people were displaced in 2012. This includes 15.4 million refugees (fleeing from their own country to another), 937 000 asylum seekers and 28.8 million internally displaced people (ie, seeking refuge within their own country). The main drivers are the wars in Syria, the Democratic Republic of Congo and Mali. Accommodating refugees places huge pressures on host countries, which tend to be low–income countries like Pakistan or Kenya. The UN has launching its biggest–ever aid appeal and estimates that half of Syria’s population will need humanitarian aid by the end of 2013. Long–term, voluntary repatriation is the best solution, but is only possible when it safe for citizens to return. (*BBC News*, 16 July 2013)

• In its annual State of the World Population for 2013, the UN urged governments to reduce teenage pregnancies by increasing girls’ human capital, rather than measures to prevent pregnancy. It found that each year, nearly 20 000 girls below the age of 18 give birth, mostly in the developing world. Girls under the age of 15 account for more than 25% of this figure, equating to 2 million births each year. As the young population grows in developing countries, this figure could rise to 3 million by 2030, and underlying causes include poverty, gender inequality, sexual violence and child marriage. Impoverished, rural and under–educated girls are more likely to become pregnant compared with their wealthier, educated, urban counterparts. Girls are more likely to experience problems if they become pregnant too soon after puberty, and around 70 000 girls in developing countries die each year as a result. Tackling teenage pregnancy could also reduce these countries’ overall high fertility rates. (*Financial Times*, 30 Oct 2013)

## ECONOMY

• A report by the UN Environment Programme and the International Fund for Agricultural Development reveal that investing in agriculture could reduce poverty in over one billion people who rely on agriculture. It states that over 80% of the food consumed in the developing world is provided by 2.5 billion people who manage small–scale farms. According to a previous study, increasing farm yields by 10% reduced poverty in Africa by 7% and in Asia by 5%. When agricultural gross domestic product (GDP) is increased by 1%, the impact on poverty is five times as great as when the GDP of other sectors is increased by 1%. Therefore investing in and supporting smallholders by providing them with growth opportunities and incentivizing sustainable farming, will further the progress towards reaching the anti–poverty Millennium Development Goals. (*UN News Centre*, 4 Jun 2013)

• Momentum is steadily building towards the creation of a new international bank by the BRICS countries. Its leaders reviewed progress at a special summit on the sidelines of the St Petersburg G20 Summit in early September, with an expected final plan at the 6th official BRICS Summit in early 2014. Initial capitalisation is expected to be US$ 50 billion with 20% in cash and 80% in guarantees. This suggests that the bank could rapidly become a major agent in development financing and reducing poverty, focusing on infrastructure and sustainable development. The drivers behind its creation are a combination of growing BRICS economic power and frustration with the slow pace of reform of World Bank and IMF, plus the lack of growth in their volume of lending. The new bank will be closely watched to ascertain its influence on changing the development financing landscape for inclusive growth. (*Oxfam.org.uk,* 7 Jun 2013)

• Talks to further liberalise trade markets between the EU and USA opened in June 2013. Mr David Cameron, the British Prime Minister, expressed hopes that a successful deal would create two million jobs world–wide, boosting global employment. Trade in goods and services between the EU and USA is worth US$ 1 trillion each year, and is the world’s largest trading relationship. It is hoped that a deal would add US$ 137 billion to the US–economy, and US$ 161 billion to the EU–economy, thus helping the EU to recover from recent economic crises. Much of current USA–EU trade is already tariff–free, but there is scope for liberalising some areas, such as financial services and airline restrictions. Talks were stalled during the US Government shutdown in October 2013, but they are scheduled to conclude at the end of 2014. Concerns have been raised that further trade liberalisation could undermine citizen and consumer safeguards on food safety, data privacy and the environment. (*Financial Times*, 8 Jul 2013)

• The UK’s overall unemployment rate has fallen throughout 2013. However, it remains stubbornly high amongst people aged 16–24, with more than one million people in this group not in employment, education or training. According to a WHO review, this is a “public health time bomb waiting to explode.” Unemployment has immediate health consequences, including a higher risk of depression and suicide, with increased risks of diseases such as cancer, heart disease and stroke in the longer–term. Prof. Sir Michael Marmot, the review leader, called for the government to look at the impact of their policies on peoples’ lives, and the impact on inequality. “Health inequality, arising from social and economic inequalities, is socially unjust, unnecessary and avoidable, and it offends against the human right to health,” he says. (*BBC News*, 30 October 2013)

• Economic growth in the Eurozone faltered in the third quarter of 2013, reducing the impact of earlier growth which saw the currency zone emerge from an 18–month recessions. It expanded by 0.1% in the third quarter, compared to 0.3% in the second quarter. German growth slipped back to 0.3%, and the French economy shrank by 0.1% after growing 0.5% during the previous quarter. Weaker exports were at the heart of these declines. The poor data from the Eurozone’s two economic powerhouses will cause concern over the durability of a wider recovery throughout the region, although there are hopes that growth will rebound in the final quarter of 2013 and throughout 2014. (*Financial Times*, 4 Nov 2013)

## ENERGY

• Stock price of Tesla Motors, Inc, the pioneering electric car producers, has skyrocketed from US$ 35.36 at the beginning of the year to nearly US$ 200 towards the end of the year. The outstanding performance can be attributed to stable demand and strong safety ratings of Model S, along with sustained innovations by the company. Tesla recently announced its plans to develop an autopilot system, which will allow the car to cover 90% of the miles driven on its own. Tesla is aiming to launch the car in 2016, although a completely self–driven car will take much longer to develop. Apart from this, Tesla is also aiming to make its car more affordable to boost sales. The company is reportedly developing a comparatively cheaper car to make electric cars accessible to the masses. (*Zacks*, 20 Sep 2013)

• The World Energy Outlook’s annual report predicts that energy prices in the USA will remain low, giving it an advantage over competitors. Any actions to reduce the impact of high energy prices should not mean decreasing efforts to address climate change, according to the report. Energy–related CO_2_ emissions are set to rise by 2035, which could lead to a long–term average temperature increase of 3.6°C, far above the 2.0°C target. As a result, the report calls for more support of renewable energy sources. (*World Energy Outlook*, 12 Oct 2013)

• In October 2013, the British government announced plans to build Britain’s first nuclear reactor since 1995. At its 1997 peak, nuclear power provided 26% of Britain’s electricity; now it is 19% and is set to fall further as older plants close. This is planned to be the first of a dozen plants built by 2030, making Britain the biggest market for new nuclear developments outside Asia. The raw materials for nuclear power are cheap, and plants produce constant, low–carbon energy – vital for meeting carbon reduction commitments. Construction costs, however, are high and require much state support. Longer–term, renewable energy is getting cheaper whereas nuclear energy is becoming more expensive due to higher safety standards. Other countries are turning away from nuclear energy, but decades of underinvestment in Britain’s energy infrastructure leave few options for meeting future energy and environmental needs. (*The Economist*, 26 Oct 2013)

• Across the USA's Midwest, 2013 was a banner year for wind energy. In May this year Warren Buffett’s MidAmerican Energy announced a US$ 1.9 billion investment in Iowa’s wind industry, which will be the largest investment of any kind in state's history. Moreover, the company Facebook announced that it would be building a new multi–million dollar data center in Iowa. This online social network explained that their decision is mainly due to the amount of power that Iowa generates from clean sources compared with neighboring states, which amounts to more than 25 percent of its energy derived from wind. (*Earth Techling*, 9 Dec 2013)

## ENVIRONMENT

• In his latest book, *Global Crisis,* military historian Geoffrey Parker examines how the frosts and famines of the 17th century’s ‘little ice age’ sparked a global wave of revolution, religiosity and disease. Without emphasising any particular country, Parker observes the patterns of widespread poverty and starvation, and how ensuing epidemics and unrest challenged the strength of the Italian states, the empire of Ming China and the unity of Great Britain, amongst others. As populations shifted, diseases such as bubonic plague and typhus crossed borders, and weakened political structures succumbed to revolutionaries and opportunists. In panic, people turned to religion with renewed passion. Mr Parker argues that the parallel crises of a diverse range of nations reflect the caustic power of climate change, as well as the human response of fundamentalism and insurgence. (*WSJ*, 31 May 2013)

• A study by the University of Minnesota found that agricultural productivity must increase by at least 60%, to provide enough food for the world’s population by 2050. Yields of key crops are only projected to grow by 38–67% by 2050, which is insufficient if actual population increases are mid– to high–range of estimate ranges. This does not take into account the possible effects of climate change on agricultural productivity. Climate and environmental degradation could be accelerated if pristine land is cleared for agriculture to compensate for the shortfall. It emphasised other ways of improving the world’s supply, such as increasing efficiency and cutting food wastage. (*The Guardian*, 20 June 2013)

• In June 2013, the world’s three biggest polluters (China, the USA and Europe) announced new carbon–reduction measures. Both China and the USA’s measures are more ambitious and far–reaching than previous policies, and the EU announced new reductions in car emissions. US and Chinese approaches are characterized by introducing some ceilings on emissions and regulations on polluting activities. Apart from China’s tentative introduction of a carbon–trading scheme in the city of Shenzhen, neither polluter tried to emulate the EU in introducing market mechanisms to control carbon emissions. Experts fear that this means pollution efforts will be ineffectual, and will not keep global temperature increases below 2°C – the maximum that most climate scientists believe to be safe. There are increasing calls for the introduction of a carbon tax, which is simpler and less vulnerable to fluctuations in emissions than current “cap and control” schemes. (*The Economist*, 29 Jun 2013)

• According to a World Bank report, environmental degradation costs India US$ 80 billion a year, or about 6% of its gross domestic product with the main culprits being air pollution, poor water supply and sanitation, and land degradation. Other surveys show that India has the world’s worst air pollution, and 23% of child deaths are attributed to environmental causes. The report showed that cutting air pollution would not interfere with India’s economic growth. Reducing large–dust particles by 30% would reduce GDP growth by 0.04% per annum, but would save an estimated US$ 47–105 billion from reduced damage to human health, plus cut CO_2_ emissions by 30–60%. (*Financial Times*, 17 Jul 2013)

• According to a survey by the European Environmental Agency, Bulgaria’s air quality is the worst in Europe, followed by Poland. It has the highest concentration of particle matter from industry, car fumes or other sources. This can cause health problems from asthma to cancer, and Bulgaria has one of the highest death rates from air pollution. Poor air quality is partly due to Soviet–era industrialisation with little attention to environmental issues, and a lack of resources for cleaning up its air. Industrial and vehicle pollution has been exacerbated by people switching to wood–burning for domestic use, as they cannot afford high energy prices. This is against a background of generally–improving air quality across Europe. (*NYT*, 5 October 2013)

## FOOD, WATER AND SANITATION

• The UN Secretary–General, Mr Ban Ki–moon warned that the world risks running out of fresh water unless global water security is improved. There are more pressures on water supplies due to increasing energy generation, whilst extreme weather events hamper natural water storage, and climate change disrupts rainfall patterns and soil moisture levels. “Under current trends, future demands for water will not be met,” he said. To put this into context, the US Geological Survey reports that although 70% of the earth’s surface is covered by water, freshwater comprises 2.5% of this total, and only 1.3% of all fresh–water is accessible as surface water. The latest UN World Water Development Report called for resources from the Green Climate Fund to be directed at the challenges faced by the water sector. (*The Guardian*, 22 May 2013)

• At the July meeting of the African Union, leaders pledged to re–prioritise agriculture in their national policies, and redouble efforts to end hunger by 2025. This is against the backdrop of strong economic growth across much of Africa failing to eradicate hunger. Across Africa, nearly 240 million people (a quarter of the population) are undernourished, and more than 40% are children under five years of age. Leaders promised to intensify efforts to increase agricultural investment and productivity to meet this goal. They recognised the importance of women in agriculture, who comprise 70% of the agricultural work–force, and their need for access to credit and land; plus the need to expand Africa’s trade partners. The final declaration did not set out any targets or cash commitments. (*The Guardian*, 2 Jul 2013)

• The World Bank’s July Food Price Watch showed that food prices reached a new high in August 2012, sparking concerns about food security and poverty. Although food prices have since slowly declined, the linkages between food, security, aid and development are still under scrutiny. To date, debate has focused on short–term food aid vs long–term capacity building and developing resilience. The development agency ACDI/VOCA says that food emergencies will always exist. Effective programmes must co–exist with longer–term development, support market conditions, and be based on local needs. Supporting local farmers and companies to adopt drought–resistant seeds, climate–smart farming techniques, and intelligent distribution systems can help build capacity and resilience. This enables local food supplies to move from areas of surplus to deficit in times of crisis. Developing agriculture in emerging economies can also provide industries in developed economies with a new market for their commodities. (*World Bank Food Price Watch*, 31 July 2013)

• Today, 2.5 billion people lack access to safe sanitation, causing serious health problems and death. Most of these deaths could be prevented by improved sanitation, along with safe drinking water and better hygiene. In 2011, the Bill and Melinda Gates Foundation launched the “*Re–invent the Toilet*” Challenge to design toilets that capture and process human waste without piped water, sewer or electrical connections, and transform waste into useful resources such as energy or fertiliser at an affordable price. So far, it has funded 16 research institutions world–wide as part of the challenge. (*The Bill and Melinda Gates Foundation*, 3 Oct 2013)

• Water is becoming the main environmental problem in China, ahead of smog, habitat destruction and food safety. Yearly, China uses 400 m^3^ of water per person, 25% of the USA level. It has 20% of the world’s population but only 7% of its freshwater supplies, complicated by having most water in the south, and half the population and most agricultural land in the north. China is rapidly using up supplies, and much water is unusable due to pollution. The World Bank put the related cost to China as 2.3% of GDP, mostly due to health damage. The government is increasing supplies to deal with water shortages, eg, canals that divert water from one part of the country to another. These projects divert water from neighbouring countries and have huge environmental impacts. Instead, experts recommend improving China’s water efficiency, recycling more water used by industry, and reducing agricultural wastage and public consumption. (*The Economist*, 12 Oct 2013)

## PEACE AND HUMAN RIGHTS

• The 2013 Global Peace Index (GPI) ranks 162 countries by their security in society, degree of militarisation and the extent of conflict. It shows that levels of peace have dropped by 5% since 2008. There has also been a shift in the nature of conflict: though a decrease in hostility between states has occurred, a disproportionate increase in internal conflicts has occurred. So what are the implications? A decrease in peace does not only affect those involved in the conflict. The global economy has suffered greatly: the GPI states that in 2012, violence cost US$ 9.46 trillion US dollars, 11% of gross world product. This is 75 times more expenditure than official assistance provided to countries in need in 2012. The findings signal a need for greater focus on peace as a prerequisite for growth and prosperity. (*The Guardian*, 11 June 2013)

• According to a report from the UNESCO Institute of Statistics and Education for All, the world is not on track to meet the Millennium Development Goal (MDG) of Universal Primary Education. In 2011, 57 million primary age children did not attend school. Despite this, international aid for basic education fell for the first time since 2002, with a reduction from US$ 6.2 billion to US$ 5.8 billion between 2010 and 2011. In this period, six of the ten largest bilateral donors reduced their basic education aid. This mainly affected low–income countries, who only received 33% of the total aid for primary schools. With only a 2% reduction in the number of out–of–school children between 2005 and 2011, the world is unlikely to meet education MDG. It therefore calls for post–2015 goals to include specific donor aid targets to ensure financing for education. (*UNESCO*, 28 June 2013)

• Recent research shows that more than one in ten men surveyed in six Asian countries (Bangladesh, Cambodia, China, Indonesia, Papua New Guinea and Sri Lanka) admitted to raping a woman who was not their partner. This rose to nearly one in four men when partners were included. Questions were worded to avoid the word “rape,” but whether the man had “forced a woman…to have sex.” Answers varied for non–partners, from 4% in Bangladesh to 41% in Papua New Guinea. Sexual entitlement was the main reason, suggesting that transforming women’s status is essential to ending the violence. More than one in seven rapists committed their first rape when they were younger than 15, and more than 50% before the age of 20, so targeting young people is also crucial. Figures were much higher in areas of recent conflict, and childhood trauma and exposure to violence may be additional factors leading men to rape. (*The Economist*, 10 Sept 2013)

• The UN Food and Agriculture Organisation’s report *The State of Food Insecurity in the World 2013* assesses world–wide levels of undernourishment, under–nutrition and progress towards the Millennium Development Goal’s and World Food Summit’s targets. It shows that by 2013, 842 million people (one in eight people worldwide) suffer from chronic hunger. Unacceptable as this figure is, it is 17% reduction from 1990–1992. If progress continues to 2015, the MDG hunger target will be nearly met. Growth must be shared to reduce hunger, and that reductions in hunger are most notable in East and South East Asia and Latin America. It noted the importance of improving agricultural productivity and increasing food production in reducing hunger and spurring economic growth. It drew attention under–nutrition and the corresponding role of nutrition–enhancing interventions to improve health, and the necessity of long term commitment to mainstreaming food security and nutrition. (*UN FAO*, 1 Oct 2013)

• The World Economic Forum’s annual Global Gender Gap Index captures and tracks the scope of gender–based gaps against economic, political, education and health criteria benchmarks, aiming to reduce gender gaps. It provides rankings that allow comparisons across countries, regions and income groups. Ranking is based on access to resources and opportunities, not availability. The Middle East, North Africa and sub–Saharan Africa have the greatest gender inequalities. There is wide variation between countries, as the Gulf States have invested heavily in female education, whereas others like the Yemen have very low levels of female education. Some sub–Saharan African countries (eg, Chad and the Ivory Coast) are near the bottom, but others (eg, Mozambique and Lesotho) perform better due to higher female labour force and political participation rates. Overall, Nordic countries have the narrowest gender gaps, and perform well compared to southern Europe. Burundi, Lesotho and South Africa all feature in the top 30 countries with the lowest gender gaps, ahead of some developed countries, eg, Japan and South Korea. (*World Economic Forum*, 30 Oct 2013)

## SCIENCE AND TECHNOLOGY

• Microbial drug resistance is a growing global health threat, but antimicrobial drug development does not offer the financial returns needed to secure investment from major pharmaceutical companies. To address this, the US Health and Human Services Department announced the payment of up to US$ 200 million over five years to GlaxoSmithKline, to enable the development of new antimicrobials for use in cases of drug resistance and bioterrorism. The US Congress also approved giving companies an additional five years’ exclusivity for successful drugs, and directed the US Food and Drug Administration (FDA) to speed up approval of new drugs. Earlier in 2013, a new European public–private partnership to develop new antimicrobials was announced. COMBACTE (Combating Bacterial Resistance in Europe) has funding of US$ 265 million. (*New York Times*, 2 June 2013)

• Bill Gates is a leading investor and supporter of the Berlin–based research network ResearchGate, which supports global research scientist collaboration. Dubbed the social network for scientists, it allows ease in exchanging scientific and experimental information, regardless of geographical location. Scientists and researchers can exchange information online and even look for potential collaborators. By allowing widespread accessibility, it aims to accelerate improvements in scientific knowledge. ResearchGate’s Chief Executive, Mr Ijad Madisch, launched the network in May 2008. Mr Gates intends the network to be a catalyst for research in his efforts to eliminate global diseases. (*Reuters,* 4 June 2013)

• In June, the Supreme Court ruled that human genes cannot be patented. The case involved Myriad Genetics, who discovered the BRCA1 and BRCA2 genes linked to increased risks of breast and ovarian cancer, thus becoming the only company to produce tests. Justice Clarence Thomas said “Myriad did not create anything,” as genes were not modified and a product of nature cannot be patented. It is hoped that this ruling will enable the test to become more widespread and affordable. It has already led to two universities and three companies offering the tests at lower prices. The Obama administration backed the ruling. (*New York Times*, 14 Jun 2013)

• Adding silver to antibiotics makes them between 10 and 1000 times more effective at fighting infections, research suggests. It could counteract the rise of drug–resistant microbes, a development that is described as both alarming and irreversible. Silver acts against Gram–negative bacteria – one of the two main types of bacteria, which are particularly difficult pathogens to treat. The research was led by Dr Jose Ruben Morones–Ramirez of the Howard Hughes Medical Institute at Boston University, and further studies will focus on testing how silver can be added to antibiotic injections or tablets for use in patients. (*BBC News*, 20 Jun 2013)

• The 2013 Nobel Prize in physiology or medicine was awarded to three researchers who explained the workings of a cellular nano–technological system. James Rothman, Randy Schekman and Thomas Südhof explained how cellular bodies called vesicles – little bubbles encased in fat – are used to carry hormones, enzymes and other items around a cell, and how they export them outside it. This transport system is vital for everything from cell division to the regulation of bodily systems through hormones. Diabetes, botulism and several neurological illnesses are partly caused by malfunctioning cellular processes, and better knowledge of how they work will lead to improved treatments. (*The Economist*, 7 Oct 2013)

## JOURNAL OF GLOBAL HEALTH's NEWS TEAM:

Rachael Atherton, Shweta Bhardwaj, Vijna Boodhoo, Kate Booth, Rachel Burge, Jenny Falconer, Alexander Fullbrook, Deirdre Halligan, Samantha Hardie, Min Ke, Ewan D. Kennedy, Lois King, Amir Kirolos, Simon McKenzie, Judith Oguguo, Diana Rudan, Victoria Stanford, Hannah Webb, Iwan Williams, Daisy Woolham, Goran Abdulla Sabir Zangana

